# P19 The role of policy and key stakeholder decision-making in the propagation, and deceleration, of resistance evolution

**DOI:** 10.1093/jacamr/dlad143.023

**Published:** 2024-01-03

**Authors:** Mineli Cooray, Tom Ashfield, Isabel Jimenez Acha, Mato Lagator

**Affiliations:** University of Warwick, Warwick, UK; Pfizer Ltd; University of Manchester, Manchester, UK; University of Manchester, Manchester, UK

## Abstract

**Background:**

Antimicrobials have an intangible value on reducing global health burden of infection, yet current valuation methods do not holistically capture the enablement of healthcare as a result of antimicrobials. A lack of incentive and subsequent investment into the novel antimicrobial pipeline demonstrates the urgent need for collaboration amongst those influencing the decision-making process, namely key stakeholders such as governmental bodies, industry and academia. Studies into resistance evolution focus almost entirely on proximate causes, such as the number of antibiotics used in clinical settings, or the understanding of evolutionary pathways to resistance under controlled laboratory conditions. Comparatively, the association between resistance evolution and policy decisions from governments, industry and regulatory bodies is poorly understood.

**Objectives:**

The primary aim was to evaluate the multifactorial associations between β-lactam development and evolution of β-lactam resistance to inform the current and future antibiotic development pipeline.

**Methods:**

To undertake this narrative report, we focussed on β-lactam antibiotics, given their clear development timeline and continuous molecular refinement to meet arising clinical and microbiological needs. As such, the evolution of resistance to β-lactams poses a significant threat to mortality, quality of life, and societal function. This report consists of six historical case studies, all of which have significantly influenced the antimicrobial resistance landscape since Fleming’s discovery of penicillin in 1928. We include lesser-known events in our narrative, such as the establishment of the National Research and Development Corporation (NRDC), which propelled the mass commercialization of penicillin, and the subsequent emergence of penicillinases. Pivotal events such as the emergence of ESBL and MBLs into clinical settings also provide insight into the historical decision-making process by key stakeholders such as governmental bodies, industry and academia. Literature reviews were conducted with a focus on the initial introduction of a particular β-lactam into the clinic and when resistance arose. Key stakeholder activities and policy decisions made during these time periods influenced the formation of case studies.

**Results:**

The case studies are as follows: (i) the influence of the NRDC in accelerated antimicrobial development; (ii) the introduction of the β-lactamase inhibitor—the influence of short-term clinical and financial benefit on prescribing behaviour; (iii) the ‘golden era’ of antibiotics—cephalosporin development and the consequences of short-term market forces; (iv) knowledge of MBL resistance prior to introduction of carbapenems in clinical settings; (v) consequences of controlled manufacturing for the global supply of piperacillin/tazobactam; and (vi) early surveillance studies and the beginning of the antimicrobial stewardship agenda.

**Conclusions:**

In each case study we provide insight into how key stakeholders can improve practices by learning from past experiences, to inform the future stewardship agenda. We built a case for the need to incorporate evolutionary microbiology into policy and industrial decision-making in order to improve the mitigation of antimicrobial resistance.

